# Poor methodological reporting in lupus clinical trials found in Cochrane reviews

**DOI:** 10.1186/ar3988

**Published:** 2012-09-27

**Authors:** CH Goldsmith

**Affiliations:** 1 Arthritis Research Centre of Canada, Vancouver, BC, Canada; 2 Simon Fraser University, Burnaby, BC, Canada

## Background

Results of randomized clinical trials depend on the credibility of the methods reporting to support study findings.

## Methods

We studied 24 trials from those in the Cochrane Database of Systematic Reviews with 'lupus' in the title and were printable. Each paper was scored by one reviewer using methodological criteria for design, allocation [1], blinding [2-4], reporting and imputation [5]. Scores used yes, no, or ? when it was unclear. Yes *n *(integer %) for all 24 papers are reported for each criterion.

## Results

### Design

Four (17%) papers had a sample size justification; 22 (92%) contained two groups and two (8%) contained three groups. Five (21%) stratified patients; yet two (8%) used stratification in the analysis.

### Allocation

Six (25%) stated random numbers generated and three (12%) blocked the balance associated with the allocation ratio; yet zero (0%) used blocking in the analysis. Six (25%) used a randomization list concealed from the person deciding patient eligibility, zero (0%) provided an audit trail for randomization, one (4%) stated randomization integrity. Seven (29%) mentioned the randomization constructed with a computer program or random number table.

### Blinding

Four (17%) stated the person deciding on the patient eligibility was blinded to block structure and eight (33%) claimed the study was double blinded, even though it was not clear who the two were; indeed one was really triple blinded! For three (12%) patient blinded, six (25%) therapy, four (17%) therapist, one (4%) other caregivers; two (8%) the outcome assessor; zero (0%) data analyst, zero (0%) manuscript writer.

### Reporting/analysis

One (4%) checked statistical assumptions, 23 (96%) provided baseline data, not all for every patient randomized. Twenty-one (88%) provided *P *values for group comparisons, four (17%) provided confidence intervals and zero (0%) provided numbers needed to treat. One (4%) specified subgroups in advance [6], six (25%) adjusted for baseline differences as one of the reported analyses. Four (17%) stated statistical software, but not version, zero (0%) provided the computer used for analyses.

### Imputation

Seventeen (71%) had missing data, yet one (4%) mentioned using last observation carried forward, zero (0%) used multiple imputation and zero (0%) mentioned impact on study conclusions [5]. Two (8%) provided a flowchart as suggested by CONSORT [7,8].

## Conclusion

Lupus trials did not report many of the methodological criteria that give papers credibility and validity to the study being reported. Reporting should be improved in future reports of studies of patients with lupus and related health problems. Possibly using the CONSORT checksheets would help make lupus papers more credible [7,8].
